# Correction: Donnelly et al. A Case of *DNAJC12*-Deficient Hyperphenylalaninemia Detected on Newborn Screening: Clinical Outcomes from Early Detection. *Int. J. Neonatal Screen.* 2024, *10*, 7

**DOI:** 10.3390/ijns11020028

**Published:** 2025-04-23

**Authors:** Colleen Donnelly, Lissette Estrella, Ilona Ginevic, Jaya Ganesh

**Affiliations:** Department of Genetics and Genomic Sciences, Icahn School of Medicine at Mount Sinai, New York, NY 10029, USA

In the original publication [1], the Informed Consent Statement was not included. The correct Informed Consent Statement appears below.

**Informed Consent Statement:** Written informed consent has been obtained from the parents to publish this paper.

The authors state that written informed consent for the publication was obtained from parents before submitting the paper and the scientific conclusions are unaffected. This correction was approved by the Academic Editor. The original publication has also been updated.
